# Neurophysiological Correlates of User Experience in Smart Home Systems (SHSs): First Evidence From Electroencephalography and Autonomic Measures

**DOI:** 10.3389/fpsyg.2020.00411

**Published:** 2020-03-19

**Authors:** Laura Angioletti, Federico Cassioli, Michela Balconi

**Affiliations:** Research Unit in Affective and Social Neuroscience, Department of Psychology, Catholic University of the Sacred Heart, Milan, Italy

**Keywords:** electroencephalography, autonomic measures, domotics, home automation, smart home systems

## Abstract

Home automation brings together technology, engineering, and user experience (UX). Within this framework, even neuroscience could be a valuable discipline to explore UX. For the first time, in the present work, some distinguishing effects of domotics on users’ cognitive and emotional behavior are highlighted by using the neuroscientific approach. In order to define possible effects of a smart home system (SHS) on UX, a neuroscientific multimethodology was adopted with the purpose of recording and confronting the neural activity (electroencephalography, EEG) and autonomic system responses of 19 individuals during a resting state (RS) baseline and the exploration of five different tech-interaction areas in a domotic environment. EEG findings showed a generalized neural activation reflected by alpha band activity while participants were exploring the tech areas confronted with the RS. The delta band was mainly present in temporo-central compared to frontal and parieto-occipital areas and was interpreted as a higher emotional activation related to the whole UX. This effect was found for the sixth tech-interaction area (i.e., bedroom) compared to the RS, and it is supposed to represent an enhanced emotional response and integration processing toward a higher multisensory interactive area. Regarding autonomic activity, an increase in heart rate (HR) was found for the bedroom area compared to the RS, thus showing a specific effect on physiological indices in this engaging tech area. The present research constitutes the first attempt to understand the user responsiveness to SHS, in terms of cognitive and emotional engagement, by adopting a neuroscientific perspective. Some high-value benefits derived from this approach will be described in light of the neurophysiological results.

## Introduction

### Home Automation

Home automation, also known as “domotics” (from the Latin *domus*, “home”), is an interdisciplinary science, which, by using informatic, engineering, and user experience (UX) insights, delivers ways to improve the quality of life inside and through the habitation (Navarro-Tuch et al., 2019). This research field has been defined as an extension of ubiquitous computing aiming at the development of ambient intelligence for smart environments (Aarts and De Ruyter, 2009; Cook, 2009). These systems are generally associated to adjectives like “smart” or “intelligent” thanks to their capacity to help and meet some user needs with a certain degree of automation (Kanemura et al., 2013). Drawing a sharp line between traditional houses and domotics is not possible: however, there are several peculiarities that help differentiate the two concepts. Smart homes are positively typified by flexibility, in the sense that modifying the configuration of the system is effort- and cost-free even after its installation, and by multifunctionality in terms of capacity to operate and control several different functions via one single device. These mentioned proprieties lead to easiness to implement innovative features, without adding new devices and also simplifying the wiring scheme and therefore enhancing security (ENEA Report: Elia and Santini, 2011). Domotics systems not only receive orders and store information; they can also discover patterns of consumption, make inferences, and therefore optimize processes. Consequently, the use of these technologies enables guaranteeing the avoidance of waste and repetition, facilitating and assisting user intentions, but also adjusting the system to new technologies and users’ specific needs.

### Neuroscience Studies on Smart Home Systems

Historically, researchers have been using qualitative methods (e.g., interviews) to explore in-depth users’ opinions regarding smart home systems (SHSs). Also, valuable evaluative studies have been conducted. They tend to focus on relations between users and SHSs, adopting a psychosocial perspective and collecting precious information on “how smart homes work in practice (routines, meanings, technology, and knowledge), including relational aspects and functionality” (Gram-hanssen and Darby, 2016). To fully understand the impact and the quality of the interaction between the user and an SHS, self-report methods are needed but may be not enough, because subjects provide only explicit and conscious information. In this sense, neuroscientific tools offer high-value insights exploring implicit neurophysiological mechanisms with real-time cognitive and emotional response data with a good balance between ecological and internal validity, also thanks to new instrumentations featuring wearable and wireless technologies (Mihajlovic et al., 2015). Neuroscience literature on home automation has previously focused on applying brain-controlled systems allowing people to control environment functions (Babiloni et al., 2009; Cincotti et al., 2010; Aloise et al., 2011). Other studies have explored the brain–computer interface (BCI), a system that is able to recognize patterns of electrical activity in the brain, through high-density electroencephalography (EEG) devices, therefore creating a new communication channel with the environment (Babiloni et al., 2007). However, to our knowledge, there may be a literature gap when bringing together neuroscience, UX, and home automation. In fact, no previous research has explored and studied the interaction between users and home automation from a neuroscientific cognitive and emotional perspective.

The present study aimed at investigating users’ neurophysiological correlates by considering EEG and autonomic responses (biofeedback) during interactions with a domotics environment. Specifically, EEG data allow the collection of information on brain activity, which ensures a deeper understanding of both cognitive and emotional processes (Aftanas et al., 2001; Balconi et al., 2015; Khushaba et al., 2013). Regarding autonomic activity, the data offer information about arousal and engagement levels; for example, heart rate (HR) activity modulations are linked to positive or negative emotions (Van’t Wout et al., 2006; Vanutelli et al., 2017a, b).

The use of a multimethodological neuroscientific approach allows exploration of the impact of both explicit (conscious correlates) and implicit (unconscious correlates) levels of users’ home automation system (Balconi et al., 2015). Specifically, in this study, the implicit and unconscious correlates were explored by using central and peripheral indices. Moreover, this approach may help in providing interesting insights on the UX as highlighted in previous exploratory contributions on emotional domotics (Angioletti and Balconi, 2019; Navarro-Tuch et al., 2019). For instance, EEG cortical oscillations can be informative of emotion processing, and different levels of investigation are possible. Firstly, regarding lateralization, the valence-specific hypothesis argues that both hemispheres process emotion, but each hemisphere is specialized for valence-specific emotion: with the left hemisphere more dominant for positive emotions and the right hemisphere for negative emotions (Ahern and Schwartz, 1979; Davidson, 1992). Secondly, different brain regions play specific roles related to the emotional process: (i) frontal and prefrontal cortex activation have been related to cognitive control over emotional stimuli and emotional behavior (Balconi et al., 2015); (ii) temporo-central areas have been involved in sensory processing (Kayser and Logothetis, 2007) and are a part of a more extended neural network responsive to the environment and social stimuli (Acevedo et al., 2014); and (iii) parieto-occipital areas are recruited by emotional visual stimuli, in particular when these stimuli are highly arousing (Lang et al., 1998).

During the interactions between an SHS and users, we expect to see a higher general activation compared to the resting state (RS) (baseline) because of higher involvement and resource allocation processes. Specifically, we expect to observe both brain and autonomic responses to an SHS in terms of: (i) a general decreased alpha brain power (increased activity) due to the increase of individuals’ attentional engagement (Dimberg and Petterson, 2000; Davidson, 2002; Balconi et al., 2014); (ii) different levels of delta band activation in response to SHS ascribed to emotion-related information processing and caused by the novelty of the stimulus and pleasantness experience for the user (Balconi and Lucchiari, 2006); and (iii) a concomitant increase of individuals’ HR response, showing emotional arousal and a certain level of engagement (Benedek and Kaernbach, 2011).

## Materials and Methods

### Participants

Nineteen healthy subjects (*M*_age_ = 25.05, SD_age_ = 3.05, age range: 18–27, *n*_male_ = 7) were recruited for the study. Inclusion criteria were normal, or corrected to normal, visual acuity and age between 18 and 38 y3ears. All participants voluntarily took part in the experiment after being informed about the study aims expressed by the informed consent. This research was conducted following the principles and guidelines of the Helsinki Declaration and was approved by the Ethics Committee of the Department of Psychology of the Catholic University of the Sacred Heart.

### Procedure

**FIGURE 1:**
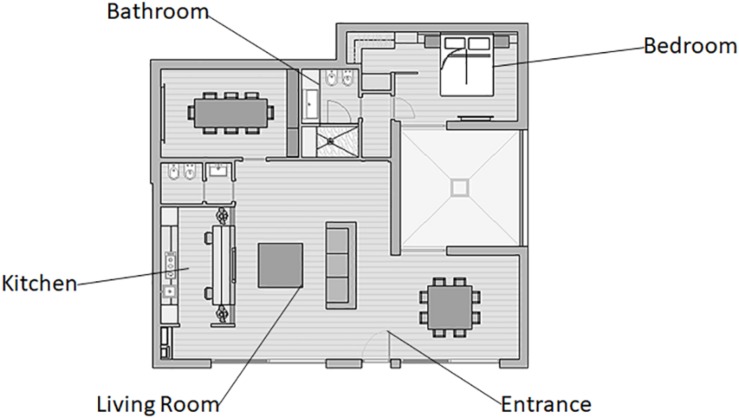
Domotic environment layout. A representation of the domotic show loft layout and the tech-interaction areas explored during the experimental phase.

The experiment took place in a home automation environment in Milan (Italy), which was the show loft owned by the tech company Duemmegi S.r.l., a developer and seller of domotics systems. Participants were asked to explore and interact with the home automation systems by using heterogeneous functions around the environment. The points of interaction were situated in the following areas of the house: hall, kitchen, living room, bathroom, and bedroom (Figure 1). The rationale for this selection depends on two aspects: (i) these areas constitute the environments present in the domotic living space and (ii) in each area, there was the installation of main devices producing effects testable by neuroscientific measures.

Each of these interactions was activated by pre-set commands on a smartphone app. The equipment was fully provided by the research team, with a short previous briefing where subjects were shown how to use the app and the interface. After the participant issued the command, each tech-interaction area produced a specific feedback eliciting novel sensory stimuli. Every interaction had a certain level of complexity in terms of response provided by the SHS. For example, the first ones consisted in a simple output presented by the system after the request of the user (e.g., turning the radio on). During the task, the SHS responses started to become more sophisticated, involving and connecting more than one device together, providing a full environmental reaction. The total exploration time for all the areas was about 30 min. The phase of exploration was preceded by the recording of a 120 s baseline in a silent zone, with subjects facing a white wall without any particular stimuli present. The first point of interaction (hall area) consisted in turning the light and the radio on. The second one (kitchen area) involved the activation of the kitchen with a hidden stove, an oven, and a wine container appearing from a normal desk. The third one (living room area) consisted in activating a multimedia projector on a screen that appeared on the wall only after the interaction started. The fourth one (bathroom area) involved the activation of a series of features such as: activation of specific lights for a shaving or makeup session, chromotherapy, and activation of a smart television. Finally, the fifth one (bedroom area) consisted in a “home bedtime mode” activation, where the blinds were closed and the home secured by locking the front door and turning off the gas and lights. The growing activation of devices in each area engaged participants in interacting with the environment. Each area involved more than one sense and provided intrinsic pleasantness in individuals, by striving to fulfill domestic needs. During the exploration, individuals’ neural activity and autonomic responses were monitored using EEG and biofeedback measures.

### Neuroscientific Measures

#### EEG

**FIGURE 2:**
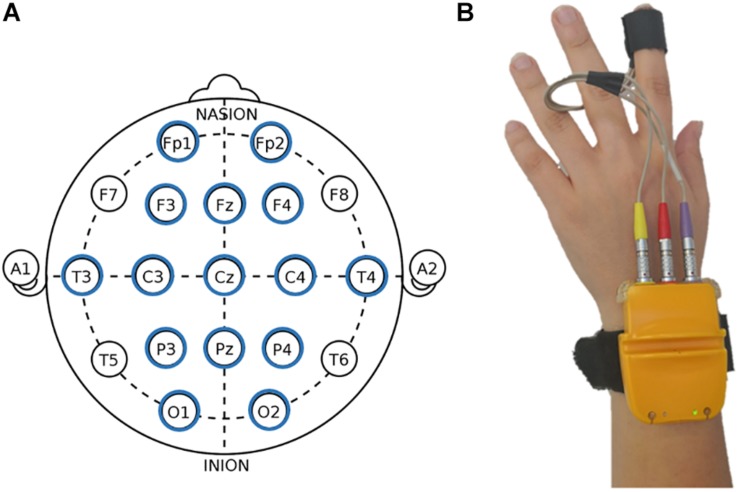
Electroencephalography (EEG) and autonomic measures display. **(A)** Fifteen-channel EEG montage adopted in the study, according to the 10/20 system of electrode placement (Jasper, 1958). **(B)** Biofeedback montage.

Electroencephalography measures were collected via a 15-channel EEG system (LiveAMP, Brain Products, Munich, Germany) with electrodes positioned over Fp1, Fp2, F3, Fz, F4, T7, T8, C3, Cz, C4, P3, Pz, P4, O1, and O2 (Figure 2A), adopting the 10/20 system of electrode placement (Jasper, 1958). An ElectroCap was used for signal recording. Data were acquired with a frequency band between 0.01 and 40 Hz and a sampling rate of 500 Hz. The electrode impedance for each individual was monitored before data collection and was kept <5 kΩ. Portions of data that presented artifacts were removed in order to increase the specificity. Ocular artifacts (blinks and eye movements) were corrected using an eye movement correction algorithm via a regression analysis in combination with artifact averaging (Sapolsky, 2004). Finally, a standard independent component analysis (ICA) analysis was applied. The EEG data were band-pass-filtered (0.1–40 Hz, 48 dB/octave roll-off), and frequency power data were computed by fast Fourier transformation (FFT) for standard frequency bands: delta (0.5–4 Hz), theta (4–8 Hz), alpha (8–12 Hz), and beta (14–20 Hz) (Keil et al., 2001).

#### Biofeedback

Autonomic measures were collected with a Biofeedback 2000x-pert system with radio module MULTI (Schuhfried GmbH, Mödling, Austria) positioned on the participant’s hand. The device was connected to a computer with Bluetooth. The system, via a sensor (4 mm diameter Ag/AgCl electrode) attached to the volar surface of the middle phalanges of the forefinger of the non-dominant hand (Figure 2B), was able to measure peripheral parameters. The recorded indices were: HR, skin conductance level (SCL), skin conductance response (SCR), pulse volume amplitude (PVA), and blood volume pulse (BVP). Specifically, for HR index, inter-beat intervals of the electrocardiogram were converted to HR in terms of beats per minute (bpm), scoring peak acceleration during the various experimental conditions. Both SCR and SCL were measured in μS: values of SCR were manually scored and defined as the largest increase in conductance during the domotics interactions. For SCL, the level of conductance as average during the conditions was considered. Moreover, for PVA and BVP, the pressure changes within the probe in the fingertip were transmitted to a personal computer (PC), where signals were band-pass-filtered (0.3–30 Hz), amplified, and stored. We considered the average of PVA variations during the home automation interaction.

### Data Analysis

For EEG data, four repeated measures multivariate analysis of variances (MANOVAs) were separately applied to the dependent measure of frequency bands (delta, theta, alpha, beta). We considered three regions of interest (ROIs) composed and obtained by averaging the electrodes in the following way: frontal (Fp1, Fp2, F3, and F4), temporo-central (C3, C4, T7, and T8), and parieto-occipital (P3, P4, O1, and O2). Also, the lateralization, in terms of the left and the right hemisphere was considered [left frontal (Fp1, F3), right frontal (Fp2, F4), left temporo-central (C3, T7), right temporo-central (C4, T8), left parieto-occipital (P3, O1), and right parieto-occipital (P4, O2)]. Analysis was carried out with the following within factors: ROI (three: frontal, central–temporal, and parieto-occipital), lateralization (two: left and right hemisphere), and area (six: baseline, hall, kitchen, living room, bathroom, and bedroom). Regarding autonomic measures, a set of four repeated measures MANOVAs was conducted for each index (HR, SCL, SCR, PVA, and BVP) as well with area (six: baseline, hall, kitchen, living room, bathroom, and bedroom) as a within factor. For all ANOVA tests, degrees of freedom were corrected by the Greenhouse–Geisser epsilon when appropriate. *Post hoc* analysis (contrast analysis for ANOVA, with Bonferroni corrections for multiple comparisons) was successively applied. The size of statistically significant effects has been estimated by computing partial eta squared (η^2^) indices.

## Results

### EEG

#### Alpha Band Activity

**FIGURE 3:**
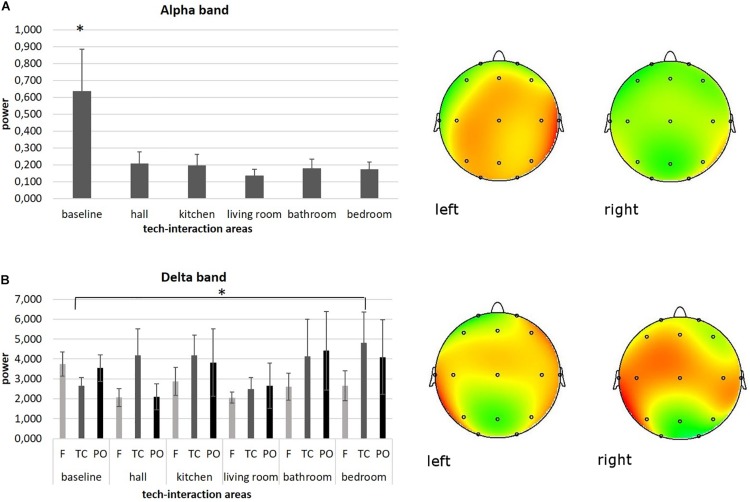
Neurophysiological EEG results. **(A)** Bar graph shows significant differences for alpha band activity between baseline and other tech-interaction areas. Bars represent ±1 SE. Stars mark statistically significant pairwise comparisons. Alpha power representation of average baseline activity (left head) compared to the average activity of the other tech-interaction areas (right head). **(B)** Bar graph shows significant differences for delta band activity in temporo-central (TC) region of interest (ROI) between baseline and bedroom area. Bars represent ±1 SE. Stars mark statistically significant pairwise comparisons. Delta power representation of average baseline activity in TC (left head) compared to the average bedroom activity in TC (right head).

As shown by MANOVA for the alpha band, a main effect for area was found [*F*(1,90) = 6.09, *p* ≤ 0.001, η^2^ = 0.28]. Pairwise comparisons revealed higher levels of alpha activity for the baseline condition (for all comparisons, *p* ≤ 0.001) compared to other areas. No other significant effects were found for other areas. Significant results are reported in Figure 3A.

#### Delta Band Activity

As shown by ANOVA for the delta band, a main effect for ROI was found [*F*(1,37) = 7.46, *p* ≤ 0.001, η^2^ = 0.27]. Pairwise comparisons revealed higher levels of delta activity in temporo-centrale (TC) regions (for all comparisons, *p* ≤ 0.001) compared to other ROIs.

Moreover, a significant interaction effect ROI × area was found [*F*(1,182) = 6.33, *p* ≤ 0.001, η^2^ = 0.26]. Pairwise comparisons revealed higher levels of delta activity in area 6 compared to baseline in TC [*F*(1,18) = 6.61, *p* ≤ 0.001, η^2^ = 0.26]. No other significant effects were found for other areas. Significant results are reported in Figure 3B.

No significant effects were found for other EEG bands (theta and beta) and for lateralization factor.

### Biofeedback

**FIGURE 4:**
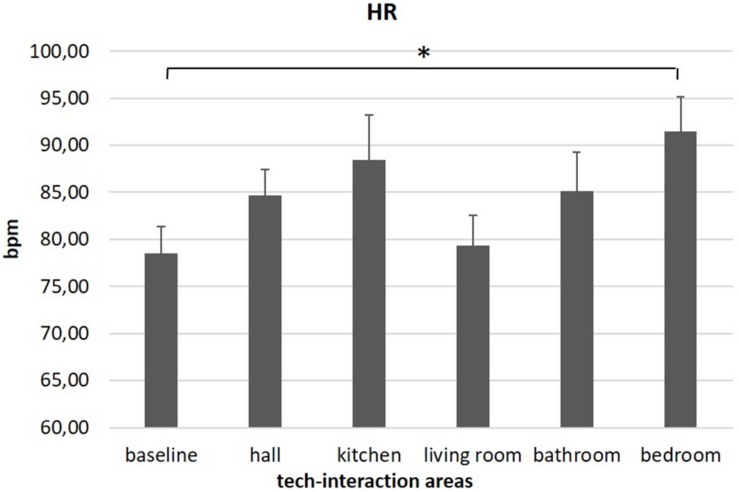
Autonomic results. The graph shows significant differences for heart rate (HR) between baseline and bedroom area. Bars represent ±1 SE. Stars mark statistically significant pairwise comparisons.

As shown by MANOVA for HR, a main effect for area was found [*F*(1,17) = 5.54, *p* = 0.031, η^2^ = 0.24]. Pairwise comparisons revealed lower levels of HR for the baseline condition compared to the sixth area (bedroom) [*F*(1,17) = 5.54, *p* = 0.18; η^2^ = 0.24]. No other significant results for autonomic measures were found for other areas. Significant results are reported in Figure 4.

## Discussion

This study provided new insights on the neurophysiological correlates of UX inside a domotic environment using neuroscientific tools. Specifically, participants explored an SHS show loft composed of five main tech-interaction areas while their central (electrophysiological) and autonomic (peripheral) system activity were collected. Tech-interaction areas coincided with the show loft’s ambients (hall, kitchen, living room, bathroom, and bedroom), and every interaction area had a certain level of complexity in terms of the response provided by the SHS: indeed, in the last areas, the SHS responses started to become more sophisticated, involving and connecting more than one device together, providing a full environmental activation. This increasing level of complexity was detected in the present sample by EEG and autonomic modulation activity. The intrinsic relationship between these two different levels of measures will be elucidated below.

Firstly, at the cortical level, an increase of alpha band oscillations was mainly found for the baseline condition compared to the other tech areas, and this result could be interpreted as a mechanism of attentional suppression when participants were at rest, followed instead by a cognitive generalized brain activation during tech-interaction area exploration. This result is in line with our expectations and with previous basic research on alpha band activity describing a decrease of alpha power with higher cortical excitability (Foxe and Snyder, 2011). Moreover, according to Ray and Cole (1985), alpha oscillations are lower for tasks requiring attention to the environment (e.g., our tech-interaction areas), than for those not requiring such attention (e.g., RS). This finding could suggest that in general, a domotic space and these tech-interaction areas have required relevant attentional demands to participants and engaged them at a cognitive level.

Recently Navarro-Tuch et al. (2019) integrated a classical methodology for emotion elicitation, that is, the International Affective Picture System (IAPS; Lang et al., 1990), in an experimental domotic environment, and theoretically discussed the emotional domotics general system and components. According to this model, UX depends on two components: the physiological and emotional variables that can be measured by multiple wearable devices such as physiological and cortical sensors. Although their experimental results will be discussed in future research articles, the authors stated that they mainly focused on physiological (autonomic) variables and facial expression analysis. On the other hand, the present preliminary findings may suggest that, in order to analyze a holistic UX in an SHS, the integration of neural information deriving from cortical oscillations’ functional meaning can be highly informative, firstly for understanding whether users are focusing their attention on the target and secondly to verify the coherence between autonomic responses and high-level complex reactions.

Secondly, an increase of delta power was found in temporo-central brain regions compared to anterior and posterior ROIs during the whole experimental phase. This effect might be interpreted as an emotional activation, perhaps related to the intrinsic pleasantness of the experience inside the domotic show loft (Grandjean and Scherer, 2008; Balconi et al., 2009). Indeed, regarding the cortical origin of delta waves during cognitive processes, it has been hypothesized that these low-frequency oscillations are associated with motivational and emotional states involving prefrontal brain structures (Knyazev, 2007). Previous basic research highlighted that the delta band depends on activity of motivational systems and participates in salience detection of emotional stimuli (Knyazev, 2007). In addition, its modulation was also found to be related to the arousing power of stimuli regardless of the valence. Therefore, it may be responsive to motivational and attentional significance of relevant emotional cues *per se* (Basar, 1999; Balconi et al., 2009; Balconi and Pozzoli, 2009). With reference to the ROI, anterior modulations of delta power are thought to mirror cognitive load related to emotional information processing, while delta increase over more posterior areas was proven to occur following stimulations that have an emotional positive/appetitive connotation (Balconi and Lucchiari, 2006; Balconi et al., 2009).

Moreover, this temporo-central delta band manifestation was significantly higher in the sixth tech-interaction area compared to the RS. The nature of the delta band as a marker of emotional relevance might confirm that this last area with its features was the most emotionally engaging. Nonetheless the role of the delta band during cognitive tasks has been previously associated with cortical inhibition of the sensory afferences that interfere with internal focus (Fernandez et al., 1995; Harmony et al., 1996, 2009; Harmony, 2013). Before, delta oscillations were shown to be implicated in the synchronization of brain activity with autonomic functions, in motivational processes associated with both reward and defensive mechanisms, in higher emotional involvement, and in cognitive processes related to attention and the detection of motivationally salient stimuli in the environment (Knyazev, 2009, 2012). Thus, a possible explanation might be that in the present study, delta activity in temporo-central brain regions reflected a condition of (i) emotional focus on specific external engaging and interacting environments and (ii) attentional orientation for novel and partially unexpected stimuli (characterizing the sixth tech-interaction area) (Fernández et al., 1998; Balconi and Lucchiari, 2006; Grandjean and Scherer, 2008). Otherwise, another possible alternative explanation could be that during the feedback provided by the sixth area, an intersensory integration involving sensory cortices in temporo-central brain regions occurs (Kayser and Logothetis, 2007). Still, given that this is the first study exploring the role of delta in complex dynamic environments, before coming to strong conclusions, caution is needed.

It is worth noticing that we did not find significant results for the high-frequency beta band and theta band, perhaps because these cortical oscillations reflect more the controlled emotion cognitive appraisal (e.g., goal conduciveness and task/goal relevance; Grandjean and Scherer, 2008), which is possibly less consistent in this exploratory study compared to the higher automatic emotional reactivity derived from delta and autonomic findings. Moreover, no significant differences were found for lateralization in the present study; therefore, no valence-specific effect can be stated by this first preliminary evidence. However of great interest would be the deepening of possible brain lateralization effects in users who explore and live in an SHS.

Lastly, HR activity was greater while participants were exploring the different tech areas compared to the baseline condition. Once again, this effect was mainly significant for one specific space of the show loft, the bedroom. This domotic area was characterized by the activation of different scenarios involving the sensory system: indeed, lights turned off slowly, and a vocal sound alerted the person on the activation of the security alarm.

In neuroscience literature, autonomic parameters are considered sensitive performance measures, and according to the “doctrine of autonomic space,” Backs et al. (2005) suggested a relation between increased sympathetic activation over time and the number of executive processes involved in the situation. Also, previous research suggested an association between HR increase and cognitive demand (Kramer, 1991; Roscoe, 1992; Backs and Seljos, 1994; Veltman and Gaillard, 1998; Brookhuis and De Waard, 2001; Wilson, 2002). Thus, it is plausible that users sensitively detected heightened cognitive load while entering the bedroom and invested additional cognitive resources to maintain a given level of arousal as demands increased. In line with this, HR increase was previously linked to an emotional arousal, involvement, or stress condition (Benedek and Kaernbach, 2011; Balconi and Vanutelli, 2015; Vanutelli et al., 2017a, b). On the whole, HR increase was interpreted as a confirmation of generalized activation at the physiological level related to the domotic environment’s cognitive and emotional effects compared to a resting condition. The qualitative observation of a higher HR for all areas compared to the baseline might confirm areas’ emotional intrinsic pleasantness engaging the participants. Regarding the effect found for the bedroom (sixth tech-interaction area), a significant HR increase was found concurrently with higher delta in TC brain regions, perhaps as a possible marker of motivational–emotional activation related to a noticeable difference featuring this area compared to other areas, that is, the vocal human sound on safety measures.

To conclude, the present study provides initial evidence about the integration of cortical and autonomic measures in healthy individuals during their UX inside a domotic environment and while interacting with a complex SHS. The interaction with different multisensory tech-interaction areas induced in participants a cortical and autonomic modulation related to increased cognitive processing and emotional engagement (specifically in temporo-central brain areas). Also, specific tech-interaction areas were found to be more engaging than others, and this could be due to their multisensory nature able to augment UX, since the effect was present only in the most complex areas. Regarding the use of these two measures (EEG and autonomic indices), they are independent on a functional level (Balconi et al., 2015), since for instance, HR is more representative of the autonomous emotional impact derived from the areas.

Despite its several advantages, this study is not without limitations, and it is necessary to be cautious with the interpretation of present results that, so far, constitute initial experimental evidence in the field. Indeed, the benefits of exploiting a multimethodology involving central and autonomic measures were discussed, although self-report scales and questionnaires were not directly related to the neurophysiological measures. Two main limitations of this study regard the absence of information derived from self-report questionnaires and subjective feelings participants experienced during their experiments. In the future, it would be necessary to collect information on the individuals’ subjective feelings in order to correlate them with EEG and autonomic measures. Moreover, no specific interaction effect was found for the gender variable, though our sample size should be increased – specifically for male participants – to generalize present results to a wider population. Lastly, this study did not take into consideration the comparison with people of different ages, although previous studies highlighted interesting positive attitudes toward SHSs so far by means of qualitative measures (Demiris et al., 2004; Meulendijk et al., 2011).

Nevertheless, future research could deepen these issues by directly comparing, for example, different developmental ages until elderly samples to investigate if (and how) the cognitive and emotional responses related to these complex systems change over time. Also, supplementary neuroscientific measures such as eye-tracking systems or functional near infrared spectroscopy (fNIRS) could be applied to explore the relation between ocular behavior and hemodynamic neural responses. Finally, explicit and subjective measures should be considered and interpreted together, to explore the interplay between covert and overt responses, as well as the role of individual factors, such as the technology’s degree of familiarity, gender, and age digital divide, but also personality components, including locus of control and motivational factors.

## Data Availability Statement

The datasets generated for this study are available on request to the corresponding author.

## Ethics Statement

The studies involving human participants were reviewed and approved by the Ethics Committee of the Department of Psychology of the Catholic University of the Sacred Heart. The patients/participants provided their written informed consent to participate in this study.

## Author Contributions

MB and LA contributed to the conception and design of the study. FC and LA wrote the first draft of the manuscript. MB, FC, and LA contributed to the manuscript revision and read and approved the submitted version of the manuscript.

## Conflict of Interest

The authors declare that the research was conducted in the absence of any commercial or financial relationships that could be construed as a potential conflict of interest.
